# Ocoxin as a complement to first line treatments in cancer

**DOI:** 10.7150/ijms.50122

**Published:** 2021-01-01

**Authors:** Aitor Benedicto, Eduardo Sanz, Joana Márquez

**Affiliations:** 1 Department of Cellular Biology and Histology, School of Medicine and Nursing, University of the Basque Country, 48940, Leioa, Bizkaia, Spain.; 2 Catalysis S.L., Toledo, Spain.

**Keywords:** Ocoxin, natural product, antioxidant, anti-inflammatory, cancer, chemorresistance, coadjuvant

## Abstract

Chemotherapy and radiotherapy are the most frequent treatment for patients suffering from malignant progression of cancer. Even though new treatments are now being implemented, administration of these chemotherapeutic agents remains as the first line option in many tumor types. However, the secondary effects of these compounds represent one of the main reasons cancer patients lose life quality during disease progression. Recent data suggests that Ocoxin, a plant extract and natural compound based nutritional complement rich in antioxidants and anti-inflammatory mediators exerts a positive effect in patients receiving chemotherapy and radiotherapy. This mixture attenuates the chemotherapy and radiotherapy-related side effects such as radiation-induced skin burns and mucositis, chemotherapy-related diarrhea, hepatic toxicity and blood-infection. Moreover, it has been proven to be effective as anticancer agent in different tumor models both *in vitro* and *in vivo*, potentiating the cytotoxic effect of several chemotherapy compounds such as Lapatinib, Gemcitabine, Paclitaxel, Sorafenib and Irinotecan. The aim of this review is to put some light on the potential of this nutritional mixture as an anticancer agent and complement for the standard chemotherapy routine.

## Introduction

Cancer patients diagnosed with primary and metastatic malignancies are treated with radiotherapy (RT) and a diverse group of chemical compounds, chemotherapeutics, which have been shown to be effective in controlling disease progression and, in some scenarios, eradicating the tumor mass 1-4. Even though new successful treatment opportunities are now being implemented in several cancer types, such as immunotherapy for melanoma, colorectal cancer and hematological malignancies among others 5-7, the chemotherapy (CT) agents remain as the first option for many malignancies and a complement for those patients receiving immunotherapy regimens 8-10. CT has been the only effective option for decades and its benefits are unquestionable. However, patients taking advantage from this treatment report a wide range of negative side effects that limit their life. One of the main goals of oncologist is to cope with the disease but providing good quality of life to cancer patients.

The use of a diverse spectrum of nutritional compounds is a common complement for patients receiving CT and RT, including antioxidants, anti-inflammatory mediators and vitamins 11-16. Antioxidants are one of the most routinely used complement, with moderately good results when dealing with treatment derived complications. To this respect, melatonin was proven effective in reducing the oral mucositis, which drives oral tissue inflammation and pain in head and neck cancer patients receiving CT and RT 17. A high spectrum of antioxidants was proven to be effective dealing with CT-induced peripheral neuropathy (CINP) in patients suffering from Colorectal Cancer. Several studies reported attenuated CINP in patients receiving N-acetylcysteine, Goshajinkigan (GJG or TJ-107), a traditional Japanese herbal extract and Jianwei Hiangqi Guizhi Wuwu Decoction (JHGWD) a traditional Chinese medicine herbal extract, while receiving CT treatment with Oxaliplatin 18-20. Furthermore, the antioxidant effect of Sodium thiosulfate (STS) seems to be positive to revert a critical side effect of cisplatin treatment, as reduced hearing loss in head and neck cancer patients was recently reported 21.

Ocoxin, also known as Oncoxin or Ocoxin Oral solution, is an orally bioactive nutritional mixture composed by antioxidant and anti-inflammatory natural ingredients (Table 1) (Catalysis S.L), rich in green tea polyphenols, such as epigallocatechin-3 gallate, glycyrrhizic acid, cinnamic acid and vitamin B6. These compounds have shown a positive effect when tested against cancer, reducing cancer stem cell markers in colon cancer and related to decreased colorectal cancer risk in humans 22-24. Some Ocoxin ingredients such as glycyrrhizinic acid and previously mentioned green tea polyphenols exert immunomodulatory and anti-inflammatory functions 25-28, which may affect significantly to the positive response to this nutritional complement. Recent evidences describe the synergic effect of Ocoxin ingredient Ascorbic acid against lymphoma when combined with immunotherapy 29, thus, reinforcing the hypothesis that Ocoxin may act sinergically when administered with routinely used cytotoxic agents.

Evidences obtained during the last decade uncover the role of Ocoxin in improving the life quality of CT receiving cancer patients. Moreover, preclinical models of different cancer types point out Ocoxin as an effective anticancer agent and a good candidate to be tested as a complement for the CT and RT routines in the clinical practice.

The aim of this review is to focus on the reported positive effects of Ocoxin during routine clinical cancer treatments and the effectiveness of using Ocoxin as anticancer compound and adjuvant for the CT and RT regimens.

## Ocoxin mitigates CT-related side effects in clinical trials

While fighting against cancer, patients receiving doses of CT have to deal with different complications related to the treatment. These complications include depression, loss of appetite and the consequent weight loss, diarrhea, interrupted treatment routine due to the adverse effects and inflammation of the oral cavity. There are several nutritional complements that help mitigating these side effects, which influence both the quality of life of cancer patients and determine the viability of the established treatment length 12. The composition of Ocoxin, rich in natural compounds with well-known antioxidant and anti-inflammatory effects, may give rise to improved CT-related response. Moreover, it is to note that Ocoxin has shown good results when administered as an adjuvant regimen in patients receiving CT. Even though there is still a lack of studies regarding Ocoxin bioavailability and metabolization, the observed benefits may encourage researchers to go into deep into these processes, in order to understand and apply this compound to a wide spectrum of patients.

## Ocoxin administration routine

The administration of Ocoxin along the different clinical studies conducted is specified in Table 2. Briefly, all patients undergoing CT and RT routines were supplemented with 50 or 75 ml oral Ocoxin from the beginning of the anticancer treatment until the end of the RT and CT cycles. In one clinical study Ocoxin supplementation started one month before the anticancer treatment and was sustained for one month after the conclusion of RT and CT routines. Ocoxin was administered for 3 weeks after the conclusion of the standard treatments in another clinical study. The Ocoxin administration spectrum and anticancer treatment implemented for each clinical study is specified in Table 2.

## Ocoxin and depression

Depression is a common undesired side effect of CT regimens. Interestingly, increased degrees of oxidative stress, which is one of the catalyzators of the antitumor effect of CT, are associated with depression and anxiety symptoms 30. It is to note that Ocoxin when administered to cancer patients improved their quality of life 31, 32, which was accompanied by an increased optimism, therefore, reducing the number of episodes of depression 31. Moreover, Kaidarova et al. (2019) described improved quality of life along with increased well-being in patients receiving Ocoxin supplementation, compared to that of patients without Ocoxin 33. It is tempting to hypothesize that the antioxidant potential of Ocoxin could be critical for this reported improvement. Moreover, this finding goes in line with previous reports on the use of a wide spectrum of compounds with antioxidant effects to treat human depression, such as resveratrol 34. Regarding to preclinical evidences, the same therapeutic effect was reported in different mice models, where a reduction in the depressive behavior was observed due to the reduction on the oxidative stress 35-37. Along with oxidative stress, inflammation has also been postulated as one the promoters of depression 38. In fact, it is related to depression observed in patients suffering from other nervous system pathologies, such as multiple sclerosis 39. One of the standard treatments to cope with depression is the use of antidepressants. These psychoactive drugs exert their effect in part through the reduction of the production of pro-inflammatory cytokines such us interleukin-1 β (IL-1β), interleukin-6 (IL-6) and tumor necrosis factor α (TNF-α) 40, 42, therefore, modulating immune responses. Interestingly, natural compounds such as curcumin and salidroside have shown antidepressant effect in stress-induced depression with reported decreased inflammatory IL-6 and TNF-α signals (42, 43], driving to immune regulation. A similar response was observed in patients suffering from other pathologies under Ocoxin regimen, reporting immunoregulatory properties and reducing TNF-α level 25. Consequently, Ocoxin may alleviate depression in cancer patients reducing the inflammatory status and the oxidative stress mediated damage.

## Ocoxin and Karnofsky index

The Karnofsky Performance Scale Index is a measurement tool to assess the functional impairment of patients. The aim of this index is to assess the prognosis of patients, which allows comparing different therapies and treatments. Petients with higher Karnofsky index show better prognosis. It is to note that Ocoxin administration along with CT resulted in 59,26% increase in Karnofsky index compared to 30,38 reported in patients receiving CT alone, suffering from different neoplasic diseases, such as head and neck, breast and uterine cervix cancer 31. In concordance, other natural compounds have shown efficacy when using as adjuvant along with CT regimens, increasing the Karnofsky index in cancer patients, such as those suffering from Non Small Cell Lung Cancer (NSCLC) 44. Therefore, this effect may be mediated by the overall improvement in health status and self-steem observed in patients receiving daily dose of Ocoxin as coadjuvant to CT.

## Ocoxin improves CT and RT related toxicity

The chemotherapeutic anticancer treatments cause a significant toxicity to patients, limiting their ability to tolerate the established routine for the treatment of the diagnosed malignancies. Ocoxin has shown a positive effect in mitigating CT-related toxicity, allowing patients to tolerate better the RT. In fact, Ocoxin reduced the number and the intensity of adverse episodes in head and neck, breast and uterine cervix cancer patients 31. These results were recently confirmed by Chon-rivas et al. (2018) in head and neck cancer 45. They reported decreased RT interruptions and lower side effects related to CT and RT toxicity. Almost 50% of the anticancer drugs approved by the FDA mediate the production of reactive oxygen species (ROS) or reactive nitrogen species (RNS) 44, therefore, affecting dramatically the function of healthy cells. Interestingly, antioxidant and anti-inflammatory properties of Ocoxin may be behind this improved tolerance to anticancer therapies.

To this respect, one of the side effects, CT-induced cognitive impairment (CICI), also known as “chemobrain” or “chemofog” is partly mediated by the ROS and reactive nitrogen species (RNS) produced by CT, inducing neuron death 46. This neuronal death might be partially inhibited by the antioxidant effect of Ocoxin, conferring the patient a better cognitive and health status.

CT-induced diarrhea (CID) is considered a reliable toxicity parameter in patients receiving CT 47. CID is a usual side effect of several CT agents such as Sorafenib and Irinotecan. With respect to Irinotecan, the most studied agent regarding to side effects, it is provoked by the intestinal cell damage through 7-Ethyl-10-hydroxycamptothecin (SN-38) metabolite. SN-38 can induce ROS in healthy cells, as reported in hepatocytes increasing ROS plasma levels of Irinotecan receiving cancer patients 48. Moreover, epithelial intestinal cells (IEC) enter DNA damage-mediated apoptosis process, which leads to ROS production 49. This ROS cause severe damage to IEC, promoting both inflammation and epithelial basal cell death leading to diarrhea. It is tempting to hypothesize that the antioxidant activity of Ocoxin may inhibit this ROS-mediated mechanism in the intestinal track, reducing CID. Moreover, Ocoxin mediated anti-inflammatory potential may reduce gastrointestinal inflammation, mitigating diarrhea symptoms, as reported using a Cyclooxigenase-2 inhibitor, Celecoxib 50.

Oral mucositis is a severe inflammation of the oral cavity leading to oral pain and ulcerative area presented in almost all patients receiving RT and in 40 % of the patients receiving CT 51, 52. This damage is partly mediated by inflammatory mediators such as IL-1β, TNF-α and inducible nitric oxidase sinthase (iNOS) 53, 54. Interestingly, Ocoxin exerts a curative effect in patients suffering from this side effect, diminishing the ulcerative area and facilitating the healing of the affected tissue 55, therefore eliminating the pain related to eating and drinking. This effect may be due to the anti-inflammatory properties of Ocoxin, which confers the ability to inhibit TNF-α upregulation, as reported in other pathologies 25. It is tempting to hypothesize that this effect may be mediated by Glucosamine present in Ocoxin, since its supplementation leads to downregulation of nuclear factor kappa-light-chain-enhancer of activated B cells (NF-κβ), which in turn decreased inflammatory mediators such as IL-1β and TNF-α 56. On the other hand, antioxidant potential of Ocoxin may block the synthesis of ROS and NOS during mucositis, in concordance with other findings reporting improved oral pathology using natural compounds with antioxidant and anti-inflammatory properties 57, 58.

In line with this observation, the supplementation with epigallocatechin-3-gallate (EGCG), one of the main ingredients of Ocoxin, has proven effective to mitigate CT and RT induced inflammatory disorders in patients suffering from other cancers 59, 60. Therefore, Ocoxin seems to improve the quality of life of patients, as reported by Kaidarova et al. (2019) in patients receiving adjuvant CT 33. Using Ocoxin as a complement for the CT regimen of gastric cancer and NSCLC patients (XELOX and paclitaxel+carboplatin respectively), they observed improved quality of life, along with increased body mass, appetite and albumin levels. Interestingly, Ocoxin almost eradicated the hepatic toxicity of CT regimens in those patients, maintaining alanine aminotransferase (ALT) and aspartate aminotransferase (AST) levels unchanged in 80 to 90% of the subjects. Moreover, clinical trials are now running for the evaluation of Ocoxin as a CT complement to confirm not only its beneficial potential to improve mucositis but also to increase patient appetite and body mass (ClinicalTrials.gov Identifier: NCT03577535).

## Ocoxin increases patient survival

To improve cancer patient's life quality represents a very significant positive effect of Ocoxin. However, this compound seems to prolong patient's life expectancy in combination with routine CT regimen. Al-Mahtab et al, (2015) reported a significant increase in terminal stage hepatocellular carcinoma (HCC) patient's life expectancy. All the patients from control group receiving CT suffering from HCC died within 2 months, while 52% of adjuvant Ocoxin supplemented patients died the first two months of the study. Interestingly, 26% of Ocoxin supplemented patients lived for another 3 months, while 21% of Ocoxin having individuals prolonged their life for 9 months 61. This observation goes in line with that reported by Uddin et al. (2018) , observing increased survival rates in Ocoxin receiving patients from head and neck, breast and uterine cervix cancer compared to control group (87,65% vs 74,68%, respectively) 31, always under CT routine. In line with these findings, clinical trials using Melatonin as adjuvant to CT resulted in 1 year increased survival rate while promoting the regression of tumors 62. The same effect was reported using melatonin in combination with cisplatin and etoposide for NSCLC, improving tumor regression and increasing 6% the 5-year survival rate 63. It is tempting to hypothesize that the antioxidant and anti-inflammatory effect of Ocoxin reverts the undesired side effects of CT, which leads to improved overall physiology of patients undergoing anticancer treatments, prolonging their life expectancy.

Immune response modulation is a common step during cancer 64, 65, and plays a critical role in disease progression. In fact, the inhibition of the antitumor immune response represents one of the hallmarks of cancer development 66, 68. It is to note that Ocoxin has an immune activation effect, which is the opposite effect mediated by both tumor and tumor-activated stromal cells in the tumor microenvironment 64, 65, 68. Therefore, the activation of the immune response, along with the antioxidant and anti-inflammatory properties of Ocoxin may lead to reported increased survival in HCC, head and neck, breast and uterine cervix cancer patients.

Which is still to be deciphered is the direct antitumor effect of Ocoxin in cancer patients. Interestingly, a significant anticancer effect has been observed in different preclinical *in vitro* and *in vivo* cancer models. These preclinical models have proven a synergic effect of Ocoxin with a wide spectrum of CT agents *in vitro* and *in vivo*, even reverting stromal-mediated chemorresistance.

## Evidences of the positive effect of Ocoxin in the liver metabolism in patients under CT regimens

One of the main organs suffering from CT routines is the liver. This organ is the responsible of metabolizing anticancer drugs, making the liver function monitoring a required measure 69. Decreased albumin levels reflect liver damage. Moreover, the National Cancer Institue (NCI) assessed liver damage based on the increased serum levels of liver enzymes ALT, AST, alkaline phosphatase (ALP), and γ-glutamyltransferase (GGT) 69. Clinical studies using Ocoxin as coadjunvant for gastric cancer revealed a significant improvement regarding liver toxicity when combined with adjuvant CT. Ocoxin supplementation led to increased serum albumin levels compared to non-supplemented adjuvant CT receiving group 32. Besides, Ocoxin abrogated hepatic-toxicity related ALT and AST enzyme increase in 92% of the patients under adjuvant CT regiment after 3 weeks of treatments, while only the AST and ALT levels of 50% of the patients from non-supplemented group remained unchanged. This reduced hepatic toxicity was further confirmed by Shumsky et al. (2019) in patients receiving Ocoxin to mitigate CT and RT related oral mucositis 55. They reported 4 and 7-fold reduced ALT and AST levels in patients under Ocoxin supplementation compared to those receiving CT and RT. It is tempting to hypothesize that increased ROS levels, which are a common product of drug-mediated hepatic toxicity 70, damage liver cells, leading to liver toxicity. Therefore, the antioxidant effect of Ocoxin may prevent hepatic tissue damage, as observed with several antioxidants, such as glyzirrinic azid, one of the components of Ocoxin 71, 72.

## Ocoxin mitigates the negative effects in hemoglobin and leukocyte counts in patients under CT regimens

CT treatments have severe impact in the hemoglobin levels of individuals receiving these drugs, leading to anemia 73. Different antioxidants have shown positive effects in hemoglobin levels during anticancer treatments 74. In line with these findings, Ocoxin supplementation reduced this side effect, diminishing hemoglobin toxicity in 80% of the patients receiving adjuvant CT. However, only 58% of patients receiving adjuvant CT treatment without coadjuvant Ocoxin showed no toxicity 32. Similar results were reported in cervical cancer and endometrial carcinoma patients receiving Ocoxin as coadjuvant for CT routines. Ocoxin mitigated the decrease in hemoglobin concentration upon treatment with adjuvant CT, RT and brachytherapy 32. Interestingly, Ruiz-Lorente et al. (2020) concluded that Ocoxin was efficient in mitigating the reduction in platelet and leukocyte counts in the blood of adjuvant CT receiving patients, compared to that of adjuvant CT receiving patients with no Ocoxin supplementation 32.

## The antitumor effect in preclinical *in vitro* and *in vivo* models of Ocoxin

As previously mentioned, there is an increasing need to find new strategies to improve sensitivity to CT and to prevent the produced side effects on patients undergoing anticancer treatments. Natural products are being implemented with promising results as coadjuvant for CT, not only for their positive effect in mitigating side effects but also for their anticancer action. To this respect, Ocoxin is a mixture of several natural compounds, which has proven benefits treating cancer individually. Several *in vitro* studies carried out on murine and human cell lines of different cancer types, such as breast, liver metastasis of colorectal cancer, acute myeloid leukemia (AML), liver, lung, pancreatic cancer and glioblastoma demonstrated that Ocoxin reduces tumor cell viability in a dose dependent manner (Table 3) 75-81.

This cytotoxicity of Ocoxin against tumor cells is triggered, in part, by increasing apoptosis and decreasing the proliferation of tumor cells 75-77. Supporting these results, different biological compounds, such as, flavonoids, green tea and curcumin among others, have demonstrated antitumor effects through the induction of growth arrest and apoptosis of cancer cells 82-84. It has been shown that Ocoxin slows down cell cycle of tumor cells by arresting cells on G2/M phase in case of breast and SCLC and increasing cell population on Sub G1 and decreasing in S phase in case of colorectal carcinoma 75, 79, 76. Supporting these observations, genistein, a soy-derived isoflavone induced cell cycle arrest at G1 and/or S phase in certain cancer cell lines and it is known to inhibit cancer cell growth through G2/M inhibition in cancer cells 85, 86. Furthermore, Cianfruglia et al. (2019) reported that curcumin extract, which has the ability to suppress cell proliferation, induced apoptosis, inhibited angiogenesis and suppressed the expression of antiapoptotic proteins, inhibited cell proliferation by G2/M cell-cycle phase arrest 87- 91. The same effect was uncovered using genistein. Genistein arrested the cell cycle of human urinary bladder carcinoma on G2/M phase by the down-regulation of cyclin A and cyclin B1, and up-regulation of Cdk inhibitor p21 92. Ocoxin seems to carry out cell cycle arrest by increasing the levels of the cell cycle inhibitor p27 protein and decreasing the cell cycle regulator cyclin D1 and the tumor suppressor pRb protein levels 75, 77, 79.

Furthermore, natural products are used as a coadjuvant therapy to improve and increase the tumor cell sensitivity to CT 93. To this regard, vincristine is used to treat acute lymphocytic lymphoma and neuroblastoma. Bahmani et al. (2018) described that *Centaurea albonitens* extract enhance the cytotoxicity of vincristine without increasing normal cell toxicity 94. Moreover, 5-fluorouracil (5-FU) is a CT used to treat several cancer types, such as, gastric, breast, liver and prostate 88-90. In this way, curcumin and green tea, among others, have demonstrated efficacy by increasing the sensitivity of cancer cells and reducing the produced cytotoxicity 98, 99. In this way, the co-treatment of curcumin and 5- FU/oxaliplatin increased their synergistic antitumor efficacy in gastric cancer by activating the caspase 3, caspase 8 and 9 expression 99. Moreover, the combination of curcumin with cisplating demonstrated a potent synergistic effect by activating caspase 3 in ovarian cancer 100. Interestingly, Ocoxin increased *in vitro* the cytoxicity of first line chemoterapeutic agents, such as, irinotecan, paclitaxel, gemcitabine, Ara C, doxorubicin, fludarabine, sorafenib, docetaxel, vincristine, lapatinib and cisplatin by increasing the apoptosis of cancer cells 75, 77-81. However, Ocoxin did not demonstrate any synergic effect with trastuzumab 75. Furthermore, Ocoxin reduced the side effects produced by chemoterapy in mice 71, which is in line with described improved health status of patients treated with Ocoxin and with several studies carried out in animal models using natural compounds as coadjuvants 101,102.

**Figure 1:**
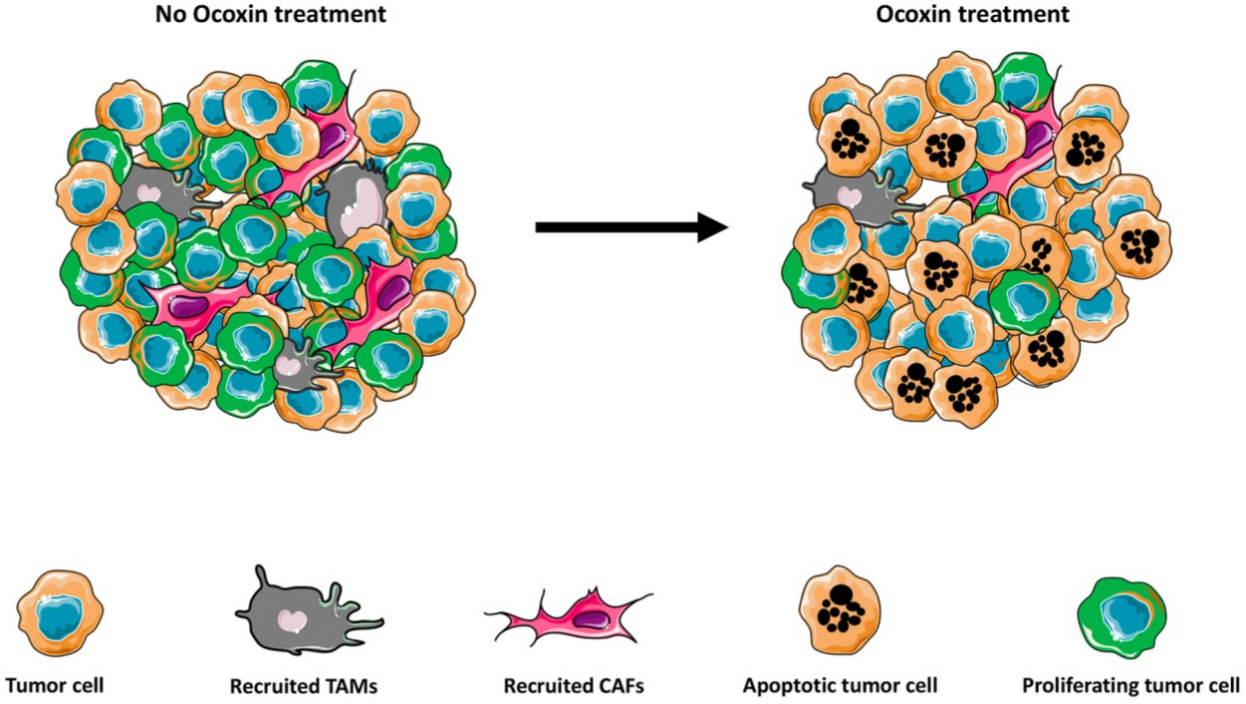
Ocoxin modulates the tumor microenvironment. Ocoxin treatment reduces the recruitment of tumor-promoting macrophages and fibroblasts into the developing tumor. Moreover, Ocoxin impairs reduces cell proliferation and increases apoptotic cancer cell death, leading to impaired tumor growth.

Nowadays one of the major challenges to treat cancer is to fight resistance to the CT. This chemoresistance may be produced by the tumor host factors 103 or factors associated with tumor-tumor microenvironment interactions 104. The implication of the tumor microenvironment on the progression of the tumor and the chemoresistance nowadays is widely recognized 105-110. The tumor microenvironment consists of tumor cells, vasculature, extracellular matrix (ECM), non-malignant cells, such as, stromal cells, tumor associated fibroblast (CAFs), immune cells and a complex signaling molecule network which include growth factors, cytokines, chemokines, and exosomes that sustain the internal connections of the microenvironment, 111,112. Interestingly, Ocoxin modulates the tumor microenvironment through several pathways (**Figure 1**).

CAFs, among others, participate in the development of tumor angiogenesis, metastasis, and chemo-resistance 112-114. Moreover, CAFS secrete collagen that regulates the chemotherapeutic drug resistance of cancer cells 115. Thus, the actual treatments could be directed not only to the tumor cells but also to block the interaction between CAFs and cancer cells to avoid the chemorresistance of the tumor. Interestingly, Ocoxin reverted the chemorresistance effect produced by CAFs on human pancreatic cells *in vitro* 81. Moreover, hepatic stellate cells (HSCs) are hepatic myofibroblast-like cells that infiltrate the tumor and develop a pro-tumoral environment 116. HSC burst liver metastasis through different mechanisms, such as, collagen deposition, immune suppressor cell recruitment and endothelial cell migration 117-120. Interestingly, Ocoxin reduced the HSC infiltration into the tumor stroma of colorectal cancer liver metastasis, which was accompanied with decreased tumor burden 76. This effect may be mediated by the reduction of inflammatory cytokines such as IL-6 and IL-1β, known to promote tumor growth, HSC mediated proliferation and increased ECM deposition. In fact, a similar reduction was reported in pancreatic cancer treated with conophylline, a plant derivate, where protumoral CAF effect was diminished 121.

As mentioned above, inflammation has been widely linked to cancer progression. In this way, one of the main source of inflammatory mediators in the tumor microenvironment are macrophages, cells of the innate immune system, which invade the precancerous tissue, and secrete factors to promote the tumor growth and metastasis 122,123. Due to the anti-inflammatory properties of some natural products, their role as new and complementary treatments is being tested. For example, green tea extract administered to mice with nonalcoholic steatohepatosis has shown to inhibit the early oncogenic response 124. In line with this results, Ocoxin regulated the cytokine milieu during liver colonization, decreased the pro-inflammatory IL1β, interferon gamma (IFNγ) and TNFα cytokine expression while reduced the macrophage infiltration into the tumor stroma and 76-78. In fact, in concordance with this observation, Ocoxin impaired the polarization of macrophages into the proinflammatory M2 phenotype in a murine glioblastoma model, along with reduced tumor growth 125. This effect may be mediated by the antioxidant effect of Ocoxin, which decreases ROS in macrophages *in vitro*, known to play an important role for M2 differentiation 126. This result is in line with the use of chlorogenic acid in glioblastoma, with a potent antioxidant properties, that showed reduced tumor growth, mediated by a repolarization of M2 macrophages into M1 127. Moreover, several antioxidants such as caffeic acid and butylated hydroxyl-anisole (BHA) have shown similar effects impeding M2 polarization. Therefore, Ocoxin impairs stromal cell recruitment affecting the generation of a proinflammatory and proangiogenic microenvironment.

**Figure 2:**
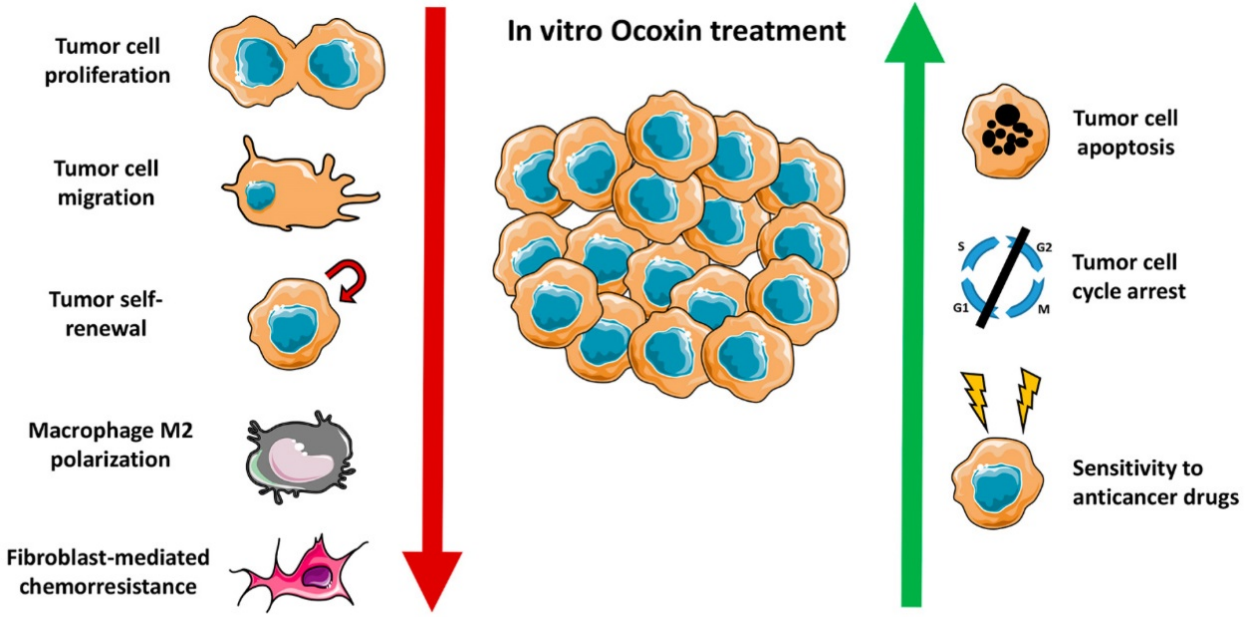
Ocoxin exerts a wide spectrum of anticancer effects *in vitro.* On the one hand, reduces tumor cell proliferation and migration, tumor self-renewal potential, macrophage M2 polarization and the protective effect of fibroblasts in tumor cells exposed to anticancer drugs. On the other hand, increases tumor cell death by apoptosis, cell cycle arrest and sensitizes tumor cells to chemotherapy agents.

In summary, Ocoxin impairs tumor development through a wide spectrum of antitumor effects (**Figure 2**). On the one hand, Ocoxin increased the cytotoxicity of anticancer agents and reduced the migration capacity of tumor cells. On the other hand, Ocoxin regulated the tumor microenviroment, by reducing the recruitment of different pro-tumoral cells and making the tumor more vulnerable and sensitive for CT.

## Conclusion and future perspectives

The efficacy of the anticancer treatments and, therefore, the subsequent healing process of patients are strongly influenced by the nutritional state 12. Taking into account that malnutrition is directly related to patient death when dealing with cancer malignancies, natural products and complements that could alleviate this physical condition should be considered as part of the treatment routine. Moreover, natural products with antioxidant and anti-inflammatory effect such as Ocoxin have been proven to be effective improving CT-related side effects and bursting antitumor effect of a wide spectrum of anticancer drugs. Therefore, if the ongoing clinical trials support these effects in patients suffering from different cancers, Ocoxin may be considered a good candidate as a complement of the routinely used RT and CT regimens.

## Authors' Contributions

AB and JM wrote the review. ES helped with the revisions of the manuscript.

## Figures and Tables

**Table 1:** Ingredient composition of Ocoxin

Average values (per 100 ml)	
Glycine	2.000 mg
Glucosamine	2.000 mg
Malic Acid	1.200 mg
Arginine	640 mg
Cysteine	204 mg
monoammonium glycyrrinate	200 mg
Ascorbic Acid	120 mg
Zinc Sulfate	80 mg
Green tea extract	25 mg
calcium pantothenate	12 mg
Piridoxine	4 mg
Manganese sulphate	4 mg
cinnamon extract	3 mg
Sodium Benzoate	100 mg
Potasium Sorbate	100 mg
Maracuya Aroma	50 mg
Sucralose	24 mg
Folic Acid	400 µg
cyanocobalamin	2 µg

**Table 2:** Clinical studies reporting significant benefits in patients receiving Ocoxin (Oncoxin) as coadjuvant for routine anticancer treatments

Reference	Cancer type	Reported benefits	Dose and regimen
31	Head and neck cancer; Breast cancer; Uterine cervix cancer	Reduced depression cases; Increased optimism; Reduced CT-related toxicity; Fewer treatment interruptions; Increased patient weight; Reduced radiation side effects; Increased survival rate	CT+RT; 50 ml Oncoxin/day for 1 year
61	HCC	Increased patient survival; Increased appetite	No other treatment; 50 ml day of Oncoxin (3 months)
45	Head and Neck cancer	Reduced RT interruptions; Reduced RT-CT toxicity	CT + RT; 75 ml day Oncoxin-Viusid (30 days prior, during treatment and 30 days after treatment)
33	Gastric cancer; NSCLC	Improved quality of life; Increased body mass; Improved hepatic function; Reduced hepatic toxicity	CT + RT; 50 ml Oncoxin/day for 20 days
55	Malignant neoplasms	Reduced hepatic toxicity; Improved oral mucositis; Increased food intake	CT + RD; 50 ml Oncoxin/day for 20 days
32	Cervical cancer Endometrial Adenocarcinoma	Improved hepatic function; Reduced hepatic toxicity; Reduced adverse effects; Improved quality of life	CT + RT + BT; 75 ml Oncoxin Viusid/daily from the beginning of CT treatment until 3 weeks after CT routine end.

**Table 3:** Preclinical studies reporting antitumor effects of Ocoxin and synergic effect with a wide variety of anticancer drugs

Reference	Cancer type	*In vitro* results	*In vivo* results
75	HER2^+^ Breast cancer	Reduced proliferation; Increased apoptosis; Cell cycle arrest; Synergy with Lapatinib	Decreased tumor volume
76	CRC	Reduced viability; Reduced migration; Cell cycle arrest	Reduced liver metastasis; Increased CRC apoptosis; Reduced proliferation; Reduced CAF infiltration
77	Acute Myeloid Leukemia	Reduced proliferation; Cell cycle arrest; Synergy with Ara C; Dexorubicin and Fluradabine	Reduced tumor volume; Reduced AML proliferation
79	HCC	Reduced HCC proliferation; Synergy with Sorafenib; Cell cycle retardation	Reduced HCC proliferation
78	CRC	Reduced CRC viability; Synergy with Irinotecan	Reduced liver metastasis; Increased CRC apoptosis; Reduced CRC proliferation; Reduced TAM infiltration; Synergy with Irinotecan regarding CRC proliferation
80	SCLC	Reduced SCLC proliferation; Synergy with vincristine and docetaxel; Increased cell death; Cell cycle retardation	Reduced tumor volume; Increased SCLC apoptosis; Reduced SCLC proliferation; Downregulation of angiogenesis-related genes *in vivo* in SCLC
81	Pancreatic cancer	Reduced cell viability; Cell cycle retardation; Synergy with Paclitaxel and Gemcitabine; Reduced fibroblast-mediated chemorresistance	Reduced tumor markers
125	Glioblastoma	Reduced GBM viabity; Reduced CSC renewal ability; Impaired macrophage polarization to M2	Reduced GBM tumor growth; Impaired macrophage polarization to M2
